# Beyond Predation: The Zoophytophagous Predator *Macrolophus pygmaeus* Induces Tomato Resistance against Spider Mites

**DOI:** 10.1371/journal.pone.0127251

**Published:** 2015-05-14

**Authors:** Maria L. Pappas, Anke Steppuhn, Daniel Geuss, Nikoleta Topalidou, Aliki Zografou, Maurice W. Sabelis, George D. Broufas

**Affiliations:** 1 Department of Agricultural Development, Democritus University of Thrace, Orestiada, Greece; 2 Institute of Biology, Dahlem Centre of Plant Sciences, Freie Universität Berlin, Berlin, Germany; 3 Institute for Biodiversity and Ecosystem Dynamics, Section Population Biology, University of Amsterdam, Amsterdam, The Netherlands; Swedish University of Agricultural Sciences, SWEDEN

## Abstract

Many predatory insects that prey on herbivores also feed on the plant, but it is unknown whether plants affect the performance of herbivores by responding to this phytophagy with defence induction. We investigate whether the prior presence of the omnivorous predator *Macrolophus pygmaeus* (Rambur) on tomato plants affects plant resistance against two different herbivore species. Besides plant-mediated effects of *M*. *pygmaeus* on herbivore performance, we examined whether a plant defence trait that is known to be inducible by herbivory, proteinase inhibitors (PI), may also be activated in response to the interactions of this predator with the tomato plant. We show that exposing tomato plants to the omnivorous predator *M*. *pygmaeus* reduced performance of a subsequently infesting herbivore, the two-spotted spider mite *Tetranychus urticae* Koch, but not of the greenhouse whitefly *Trialeurodes vaporariorum* (Westwood). The spider-mite infested tomato plants experience a lower herbivore load, i.e., number of eggs deposited and individuals present, when previously exposed to the zoophytophagous predator. This effect is not restricted to the exposed leaf and persists on exposed plants for at least two weeks after the removal of the predators. The decreased performance of spider mites as a result of prior exposure of the plant to *M*. *pygmaeus* is accompanied by a locally and systemically increased accumulation of transcripts and activity of proteinase inhibitors that are known to be involved in plant defence. Our results demonstrate that zoophytophagous predators can induce plant defence responses and reduce herbivore performance. Hence, the suppression of populations of certain herbivores via consumption may be strengthened by the induction of plant defences by zoophytophagous predators.

## Introduction

Plants employ a series of constitutive or inducible defences against herbivores. Induced defences are elicited by damage-associated [1] and herbivore-associated compounds, for example compounds in the oral secretions of the feeding herbivores [2, 3]. Moreover, oviposition by herbivorous insects can also induce plant defences [4]. Plant defences can be directly aimed at the herbivore and can consist of toxins or anti-digestive proteins that reduce herbivore performance [5, 6]. Plants can also defend themselves indirectly by releasing specific volatile signals that attract natural enemies of herbivores or by arresting natural enemies by providing food or shelter [7–9].

Besides their defensive function for the plant, responses to herbivory mediate interactions among herbivores attacking the same plant [5, 10, 11]. Depending on the forthcoming herbivore, herbivores sharing the same host plant at different times may interact through the induction or suppression of plant defences, resulting in increased plant resistance or susceptibility [5, 12, 13]. Negative cross-talk between different plant signalling pathways, such as the jasmonate (JA) and salicylate (SA) mediated pathways, has been shown to regulate several herbivore interactions (e.g. [14, 15, 16]).

Whereas plant-mediated interactions among herbivores have been relatively well studied, the effects of phytophagy by omnivores on herbivore performance through the induction of plant defences have not been investigated so far. This is surprising because many natural arthropod predators are omnivores. Omnivory ranges from occasional phytophagy by arthropods with a predatory life style (zoophytophagous arthropods) to occasional carnivory by arthropods with a predominantly phytophagous life style (phytozoophagous arthropods) (e.g. [17, 18]) and the few available studies on the role of omnivores in plant-herbivore interactions are restricted to the latter (e.g. [19, 20–24]). So far, oviposition of the predatory bug *Orius laevigatus* was shown to increase tomato resistance against feeding by the thrips *Frankliniella occidentalis* [25]. In addition, Pérez-Hedo et al. [26] have shown that exposing tomato plants to the mirid bug *Nesidiocoris tenuis* resulted in the activation of the absisic acid (ABA) and jasmonic acid (JA) signalling pathways and as a consequense these plants were less attractive to the whitefly *Bemisia* tabaci, but more attractive to the parasitoid *Encarsia formosa* compared to unexposed plants.

Here, we tested whether the zoophytophagous predator *Macrolophus pygmaeus* (Rambur) affects its prey through the induction of plant responses. This predator is used for the biological control of several greenhouse pests such as whiteflies, aphids and spider mites [27]. Unlike *N*. *tenuis*, which is known to produce visible symptoms on tomato plants even requiring the recruitment of pest control methods by growers in certain cases, *M*. *pygmaeus* use in biocontrol is generally considered as safe [27] and plant damage by *M*. *pygmaeus* in crops were reported only once in a survey with high predator numbers and low prey availability [28]. We exposed tomato plants to this mirid bug for a few days and investigated the performance of two of its prey species, the spider mite *Tetranychus urticae* Koch and the whitefly *Trialeurodes vaporariorum* (Westwood), on these plants after the removal of the predators. As we found that spider mites were negatively affected by the previous presence of *M*. *pygmaeus*, we also tested whether this effect (1) depended on predator density, (2) was restricted to the leaf tissue that had been exposed to the predator, (3) depended on the predator developmental stage, and (4) was a transient or persistent phenomenon. Furthermore, we assessed the levels of activity of proteinase inhibitors (PI), a compound involved in the direct defence of plants against herbivores, both in leaves that were exposed to the predator or unexposed leaves of exposed plants. We also analysed transcript accumulation of the tomato *PI-I* and *PI-II* genes, marker genes commonly used as indicators of JA-related defences, in leaves of the same plant exposed or not to *M*. *pygmaeus* in comparison to corresponding leaves of plants without exposure to this zoophytophagous predator.

## Materials and Methods

### Ethics Statement

This study did not involve any endangered or protected species. No permission was required to collect the herbivores since these are well-established at the collection sites. The predator species studied was purchased from the biocontrol industry. No permission was required to maintain the colonies in the laboratory. This study did not involve any field trial.

### Plants

We used tomato plants (*Solanum lycopersicum* L., cv. Ace 55 (Vf)) for all experiments, and cucumber plants (*Cucumis sativus* L., cv. Ginga F1, Geostore SA) and bean plants (*Phaseolus vulgaris* L.) to rear whiteflies and spider mites, respectively. Tomato and cucumber plants were grown from seeds in round plastic pots (Ø 15.5 cm) with soil (Klasmann-TS2) in climate chambers (25±2°C, 16:8 LD, 60–70% RH) and they were fertilized (1 g/l) once a week (N-P-K, 20-20-20). Bean plants were grown in a soil-perlite mixture (1:3) in rectangular plastic pots (30×20×30 cm). Plants were watered every other day.

### Predators and herbivores

The culture of *M*. *pygmaeus* was established with adults of the commercially available product MIRICAL (Koppert B.V. Berkel en Rodenrijs, The Netherlands). Adults were maintained in plastic cages (47.5×47.5×47.5 cm, BugDorm MegaView Science Co., Ltd.) on young tomato plants (two weeks old) at 25±2°C, 16:8 LD, grown in small plastic cups (100 ml). Ample *Ephestia kuehniella* Zeller eggs and bee pollen were provided as food. To obtain either fifth instar nymphs or adults of similar age for the experiments, adults (mixed sexes) were transferred to healthy plants in cages similar to those used for the rearing, and females were allowed to lay eggs for 48 hours. Upon nymph enclosure, tomato plants, moth eggs and bee pollen were provided as food for the predators.

Spider mites (*T*. *urticae*), originating from a population sampled in a tomato field near Alexandria (Northern Greece), were reared on detached bean (*P*. *vulgaris*) leaves, placed on water-soaked cotton wool in plastic trays. The trays were kept in a climate room at 25±1°C, 16:8 LD. Fresh bean leaves were provided every three days and the trays were filled with water to maintain leaf vigour. For the experiments, spider mite females of the stock rearing were transferred to detached bean leaves, placed on soaked cotton wool in plastic trays, to lay eggs for 24 hours at 25±2°C, 16:8 LD. The next day, females were removed and the leaves were maintained under the same conditions for 16 days until adult female spider mites emerged. Females, 2–4 days into adulthood, were used in the experiments.

Whiteflies (*T*. *vaporariorum*) were reared on 4–5-week-old cucumber plants (*C*. *sativus* L., cv. Ginga F1, Geostore SA) in plastic cages (47.5 × 47.5 × 93.0 cm, type 44590F, BugDorm, MegaView Science Co., Ltd.) at 25±2°C, 16:8 LD. For the experiments, 400 females from the stock cultures were transferred to a cage with two clean cucumber plants and allowed to lay eggs for 2 days. Subsequently, adults were removed with an aspirator and 5–10 day-old females (approx. one month after egg deposition) were used for the experiments.

### Performance of whiteflies and spider mites on tomato plants exposed to *M*. *pygmaeus*

To test whether the exposure of plants to *M*. *pygmaeus* can have plant-mediated effects on its herbivores, we used a density of a well-established population. Three- to four-week-old tomato plants were placed in pairs inside insect tents (60 × 60 × 60 cm, type 2120F, BugDorm, MegaView Science Co., Ltd.), which contained 25 young (5–10 days old) *M*. *pygmaeus* females. Control plants were placed inside tents without predators. The omnivores could freely move, walk, feed and lay eggs on the plants. After 4 days, predators were removed and all plants (*M*. *pygmaeus* exposed and control plants) were transferred to a climate room where they were randomly arranged on shelves. Two or three apical leaflets (Lf2-Lf4) of the second to fourth primary leaves, L2-L4, were either infested with one whitefly female (5–10 days old) or 10 young spider mite females (2–4 days old) for another four days, respectively. Using a mouth aspirator, CO_2_ anaesthetized whiteflies were transferred to clip cages (Ø 2.5 cm), which were fixed on the abaxial surface of two apical leaflets (Lf2-Lf4) of three leaves L2-L4 (six clip cages per plant). Groups of 10 spider mites were transferred using a paintbrush to each of 3 corresponding apical leaflets, where they were confined with barriers of lanolin paste surrounding the petiolule of the leaflet (90 spider mites per plant). After 4 days, all treated leaflets were inspected for the number of eggs and live adults. The number of live spider mites on each leaf was recorded. This experiment with spider mites and whiteflies was repeated in two blocks in time with 8 plants per treatment in each of the blocks. To investigate whether the leaves exposed to the herbivores differed between control and *M*. *pygmaeus* pre-exposed tomato plants in levels of defence proteins, leaves L2-L4 were harvested for analysis of PI activity (leaflets Lf2-Lf4 were pooled for each leaf) after the 4-day-exposure to the omnivore under the same experimental setup as mentioned above.

Since no effects of the previous presence of the predator were recorded on whitefly performance, all subsequent experiments were conducted with spider mites only.

### Effect of within-plant location, density and developmental stage of *M*. *pygmaeus*

To investigate whether plant exposure to *M*. *pygmaeus* resulted in changes in the exposed leaflet, an apical leaflet (Lf2 of leaf L2) of 3–4 week-old tomato plants was exposed to young (1–2 days old) *M*. *pygmaeus* females enclosed in clip cages (Ø 2.5 cm), attached to the abaxial surface of the leaflet for 4 days, whereas control plants received empty clip cages at the corresponding leaflet. One or five females were used to test whether effects were density-dependent. The predators were anaesthetized with CO_2_ before being transferred to the cages. After 4 days, *M*. *pygmaeus* females and clip cages were removed and ten spider mite females (2–4 days old) were confined on the same leaflet without access to the leaf surface that had been exposed to the omnivores by applying lanolin barriers to the petiolule and around the circular area (Ø 3 cm) where the clip cage had been located. Control plants received similar lanoline barriers. Spider mite females were allowed to feed and lay eggs for 4 days. Subsequently, the number of eggs and live females was recorded. This experiment was repeated in three blocks in time, with each block consisting of 11–14 plants per treatment (total of 36–38 plants per treatment). Since spider mite performance was significantly affected on leaves with prior exposure to 5 *M*. *pygmaeus* females, one leaflet (Lf2) of leaf L2 of exposed and unexposed control plants in another block of the same experimental setup was harvested for analysis of PI activity and expression of tomato *PI-I* and *PI-II* genes.

Plant-mediated systemic effects of predator exposure on spider mite performance were studied by exposing a complete leaf (L2) of three- to four-week-old tomato plants to *M*. *pygmaeus* for 4 days and then recording the number of eggs and survival of spider mites, as described above, on 3 apical leaflets (Lf2, Lf3 and Lf4) of leaf L5. Leaf L2 of all experimental plants (including control plants) was enclosed in a transparent plastic bottomless cylindrical cup (0.5 l) covered with a fine nylon mesh (mesh width 160 × 160 μm) in order to ensure proper ventilation within each cup. At the bottom opening of each cup, we attached a sleeve-like fine cloth which was trussed around the petiole of the enclosed leaf L2 with a thin thread. Each cup was supported by a wooden stick (25 cm). Predators were transferred through a small opening in the side-wall of each cup using an aspirator. The plants were exposed to either one or five young adult females (1–2 days old) or ten fifth instar nymphs of *M*. *pygmaeus* to determine whether the induction of plant responses depended on predator stage. The numbers of predators used are in a range that may be encountered in the field (Pappas M.L., personal observation) and have been reported on experimental plants after *M*. *pygmaeus* was relased as biological control agent [29]. To investigate whether the plant-mediated effects of *M*. *pygmaeus* on spider mite performance are transient or long-lasting, plants were either infested with spider mites for 4 days immediately after the removal of the predators or 4 days later. The different predator densities with and without delay after *M*. *pygmaeus* exposure were run in separate blocks through time, always including a set of unexposed control plants (9 to 11 plants per treatment combination). PI activity and the expression of *PI-I* and *PI-II* genes was analysed in the L5 leaf of another block of plants (9–10 per treatment) with leaf L2 previously exposed to 5 females or 10 nymphs of *M*. *pygmaeus* and in the corresponding leaf of unexposed plants. A pooled sample of leaflets (Lf2, 3, 4) of L5 was harvested either directly after the 4-day exposure to the predator or after a time lag of 4 days.

### Persistence of plant-mediated effects

We tested whether exposure of young plants to *M*. *pygmaeus* affected spider mite performance two weeks after the exposure ended. Two-week-old tomato plants (with only the first two primary leaves expanded) were exposed to two young virgin *M*. *pygmaeus* females in small insect cages consisting of a metallic, cylindrical frame (15.5 cm in diameter, 30 cm in height) placed directly on the soil of each pot and covered with a fine mesh. Plants of the control treatment were kept in cages without predators. For this experiment, we used young (1–2 days old) virgin females to minimize potential effects of oviposition, and the number of *M*. *pygmaeus* females was reduced from 5 to 2 since these plants were much smaller than those in previous experiments. After 4 days, the predators were removed and the plants were maintained in a climate room for another 14 days before 15 spider mite females (2–4 days old) were transferred to the three apical leaflets of leaf L3 of all plants (45 female mites per plant). Lanolin paste was applied to the petiolule of each leaflet as described above, and the number of eggs laid and live females were recorded after four days. For each treatment, 8 tomato plants were used in two blocks in time.

### Analysis of PI activity

Trypsin proteinase inhibitor activity was analysed with a photometric assay after Bode et al. [30]. Leaf samples were frozen in liquid nitrogen directly after harvesting and kept at -60°C or below until extraction and analysis. Frozen leaf samples were ground thoroughly in liquid nitrogen using mortar and pestle. Subsequently, 100 mg of the material was transferred to 2 ml screw-cap tubes and 500 μl extraction buffer (50 g PVPP, 18.6 g Na_2_-EDTA, 5 g diethyldithiocarbamate, 2 g phenylthiourea in 1 l 0.1 M Tris-HCl buffer, pH 7.3) was added while the tubes were kept on ice. Subsequently, the samples were homogenized twice for 20 sec at 4.5 m/s with 900 mg lysing matrix D in a FastPrep-24 instrument (MP Biomedicals, USA), and were then centrifuged (15 min at 14,000 g at 4°C) and the supernatant was centrifuged again. For each triplicate of a sample, 20 μl was added to 30 μl 0.1M Tris-HCl buffer containing 2.5 μg trypsin (bovine trypsin proteinase, Sigma-Aldrich) per well of a 96-well plate on ice and mixed thoroughly. After plate incubation at 37°C for 5 min (to enable proteinase inhibitors to interact with the trypsin), 20 μl of a BANA solution (3.1 mg/ml N-benzoyl-DL-arginine-b-naphtylamid, Sigma-Aldrich) in DMSO (dimethyl sulfoxide, Sigma-Aldrich) was added to each well, mixed and the plate was subsequently incubated at 37°C for 20 min (for the proteinase reaction). Subsequently, 100 μl of stop solution (2% HCL in EtOH) was added and the background absorbance was read in a UV/Vis spectral photometer (Multiskan GO, Thermo Scientific) at 550 nm. Subsequently, 100 μl of 0.06% p-dimethylaminocinnamaldehyde (Sigma-Aldrich) in EtOH was added to each well, and the absorbance was measured again at 550 nm after a 20-min-staining period at room temperature. The mean of the triplicates was used to calculate PI activity in the leaf extracts according to a linear standard curve of a dilution series (1.53, 1.22, 0.918, 0.612, 0.306 M) of soybean trypsin inhibitor (Sigma-Aldrich) that was run on each plate. The PI activity is expressed per mg of protein extracted. The protein content of each extract was determined with a Bradford assay: In triplicates, 10 μl of a 51-fold dilution of the leaf extracts was added to 200 μl 1x Roti-Quant (Carl Roth, Germany) in a microwell plate and read at 595 nm in the spectrophotometer. The protein content was determined according to the linear standard curve of a dilution series (0.25, 0.125, 0.063, 0.033, 0.017 mg/ml) of bovine albumin (Sigma-Aldrich) that was run on each plate.

### RNA extraction and analysis of PI gene expression

Of leaf samples that were obtained as described above, 100 mg was used to extract the total RNA using the NucleoSpin RNA Plant kit (Macherey-Nagel, Germany) according to the manufacturer’s instructions with minor modifications to increase yield: Instead of 350 μl, 700 μl of the RA1 buffer (with dithiothreithol as reducing agent) was used for initial lysis and the final RNA was digested with DNase I (Fermentas, Thermo-Scientific; USA) according to the manufacturer’s instructions. Subsequently, the RNA was precipitated overnight with isopropanol at -20°C and washed with EtOH. The RNA was adjusted to 200 ng/μl and before cDNA synthesis RNA extracts of 3/2 plants (for local and systemic leaf samples respectively) were pooled to generate 5 biological replicates. For cDNA synthesis a reverse transcription core kit (Eurogentec, Belgium) was used according to the manufacturer’s instructions. SYBR Green I based real-time PCR was performed in triplicates using a qPCR kit without ROX (Eurogentec, Belgium) on a Stratagene Mx3005P qPCR instrument (Agilent Technologies, Santa Clara, California, USA) for *Solanum lycopersicum PI-I* (Gene bank: K03290; primers: 5'-GGAATTTGACTCTAACTTGATGTGCGAAG-3'; 5'-TTCCTTAGCAAGCTTTGTTGGTACAC-3') and *PI-II* (Gene bank: K03291; primers: 5′-GGATTTAGCGGACTTCCTTCTG-3′ and 5′-ATGCCAAGGCTTGTACTAGAGAATG-3′) genes. The tomato ubiquitin gene was used as a reference gene (Gene Bank: X58253; 5′-GCCAAGATCCAGGACAAGGA-3′ and 5′-GCTGCTTTCCGGCGAAA-3′). *PI* gene expression relative to ubiquitin was calculated after adjusting for the PCR efficiencies for each gene (as determined using linReg, J.M. Ruijter, The Netherlands).

### Statistical analysis

The effect of plant exposure to *M*. *pygmaeus* on whitefly performance was assessed with a Generalized Linear Mixed Model (GLMM), with a Poisson or a binomial error distribution (function glmer in library lme4) for the number of whitefly eggs per plant and proportions of live individuals per plant, respectively. Effect of treatment on spider mite performance was assessed with a GLMM, with a negative binomial and a binomial error distribution for the number of spider mite eggs per plant and proportions of live individuals per plant, respectively. In these models, treatment was specified as fixed factor and experimental block in time and replicate (plant) as random factors.

The effect of exposing entire plants to *M*. *pygmaeus* females in tents on log-transformed PI activity data was assessed with a Linear Mixed Model (LMM; function lmer in library lme4). Treatment was considered as a fixed factor and leaf nested within plant as random factors. Log-transformed transcript accumulation and PI activity data for the rest of the experiments were analysed using a Generalized Linear Model (GLM) with a Gaussian error distribution and treatment as a fixed factor.

Contrasts among treatment levels were assessed with Tukey comparisons (function glht in package multcomp). All statistics were performed with R software, version 3.1.2; [31].

## Results

### Performance of whiteflies and spider mites on tomato plants previously exposed to M. pygmaeus

**Fig 1:**
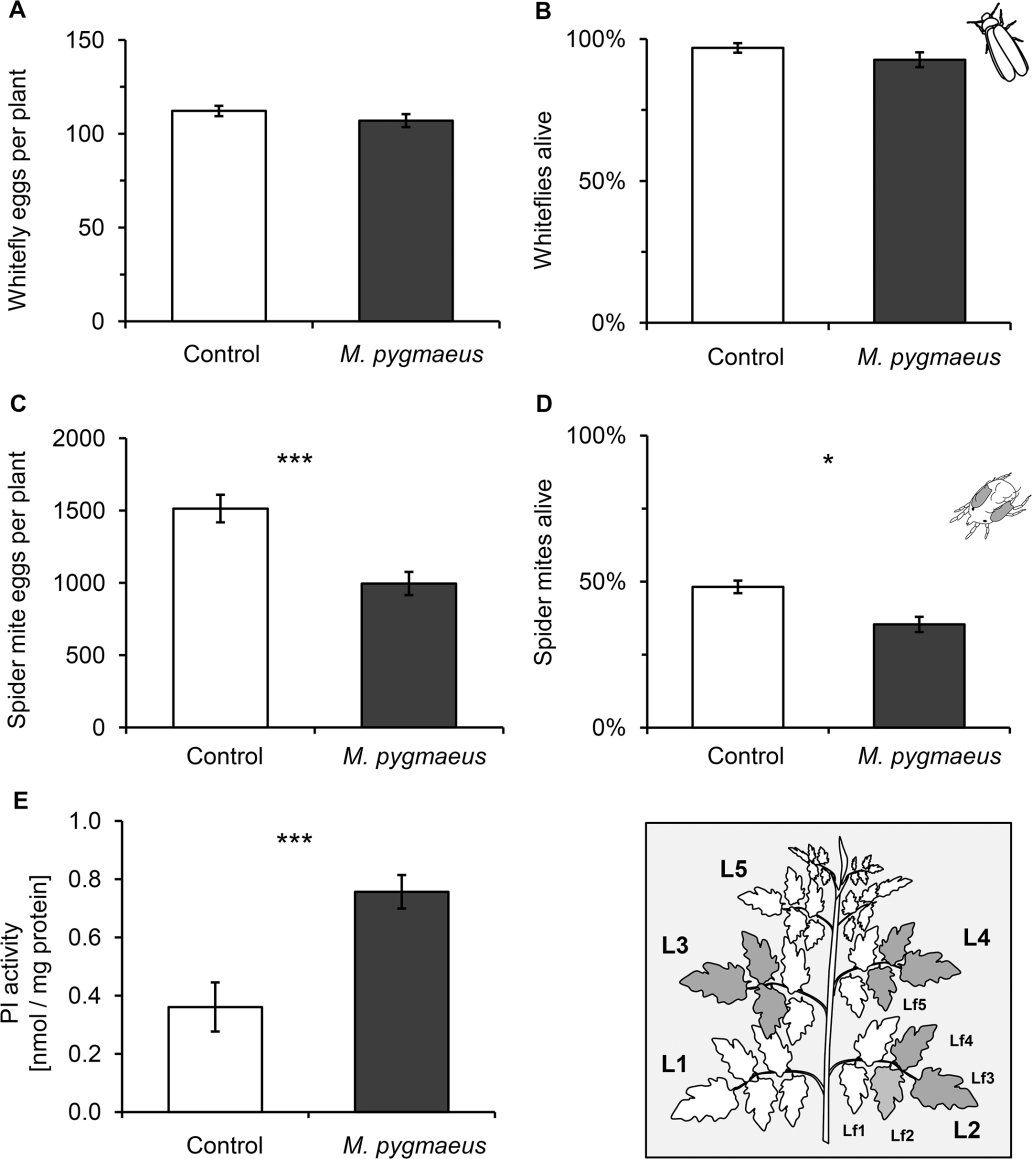
Effect of exposure of tomato plants to *Macrolophus pygmaeus* on herbivore performance. Groups of two tomato plants were either exposed to 25 *M*. *pygmaeus* females for 4 days (dark bars) or not (white bars). Mean +/- SE (N = 16) of (A) the number of eggs per plant and (B) survival of 6 adult female whiteflies per plant (2 per leaf L2-L4 on two apical leaflets (Lf) 2, 3 or 4 depicted in inset and (C) number of eggs per plant and (D) proportion of alive female spider mites out of 90 adult female spider mites per plant (10 per Lf2-4 of L2, L3 and L4) recorded on these plants after 4 days (inset). (E) Activity of proteinase inhibitors (PI) in leaf extracts (averages of leaves L2-L4 of pooled samples from Lf2-4, N = 8 plants). Significant differences between exposed plants and control plants are indicated by asterisks following a GLMM or LMM (PI data): *P* < 0.05 (*), *P <* 0.001(***).

The number of eggs and the survival of whiteflies were not significantly affected by previous exposure of entire tomato plants to *M*. *pygmaeus* (Fig 1A; GLMM; eggs/plant: χ^2^ = 1.92, df = 1, *P* = 0.1661 and Fig 1B: GLMM; whitefly survival: χ^2^ = 1.73, df = 1, *P* = 0.188). In contrast, such exposure did reduce the number of spider mite eggs per plant and the proportion of mites found alive on treated compared to untreated control plants (Fig 1C; GLMM; eggs/plant: χ^2^ = 16.46, df = 1, *P* < 0.001 and Fig 1D; GLMM; mite survival: χ^2^ = 4.88, df = 1, *P* = 0.027). Proteinase inhibitor activity levels after a 4-day exposure to *M*. *pygmaeus* were on average 80% higher than in unexposed plants (Fig 1E; LMM; χ^2^ = 21.25, df = 1, *P <* 0.001). Thus, tomato plants that were previously exposed to *M*. *pygmaeus* had an increased resistance to spider mites but not to whiteflies.

### Effect of within-plant location, density and developmental stage of *M*. *pygmaeus*

**Fig 2:**
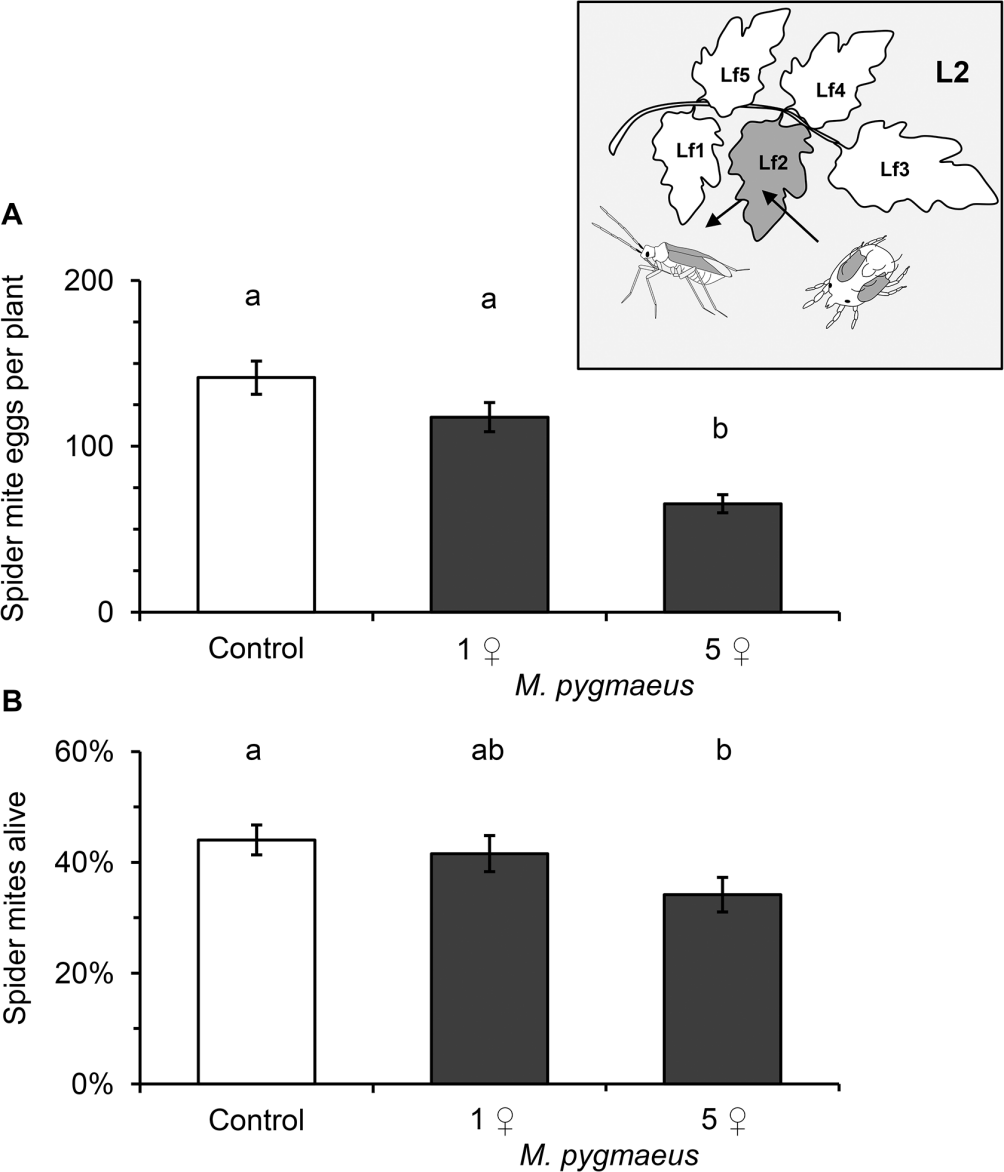
Local plant-mediated effects of *Macrolophus pygmaeus* on *Tetranychus urticae* performance. *M*. *pygmaeus* females were enclosed in clip cages on the abaxial side of the second oldest leaf (L2) at a defined leaflet (Lf2) for 4 days before 10 young *T*. *urticae* females were kept on the same leaflet without access to the area pre-exposed to *M*. *pygmaeus* (inset). (A) Number of eggs per plant and (B) proportion of alive *T*. *urticae* females on the same leaflet that was previously exposed to 1 (38 plants) or 5 (36 plants) female predators (dark bars) or the corresponding leaflets of (37) control plants (white bars) (mean +/- S.E., plants, in three blocks). Significant differences between exposed plants and control plants are indicated by different letters by Tukey contrasts following a GLMM.

We further characterized the plant-mediated effect of the omnivore on spider mites and found that it was dependent on the predator density (GLMM; eggs/plant: χ^2^ = 38.10, df = 2, *P* < 0.001, mite survival: χ^2^ = 9.07, df = 2, *P* = 0.011). Whereas prior exposure to 5 adult females reduced the number of eggs when spider mites were kept on the same leaflet by more than half, only a non-significant tendency in the same direction was observed when this leaflet was previously exposed to a single *M*. *pygmaeus* female (Fig 2A). Similarly, the proportion of spider mites found alive was only significantly reduced on leaves exposed to 5 but not 1 adult *M*. *pygmaeus* compared to unexposed control plants (Fig 2B).

**Fig 3:**
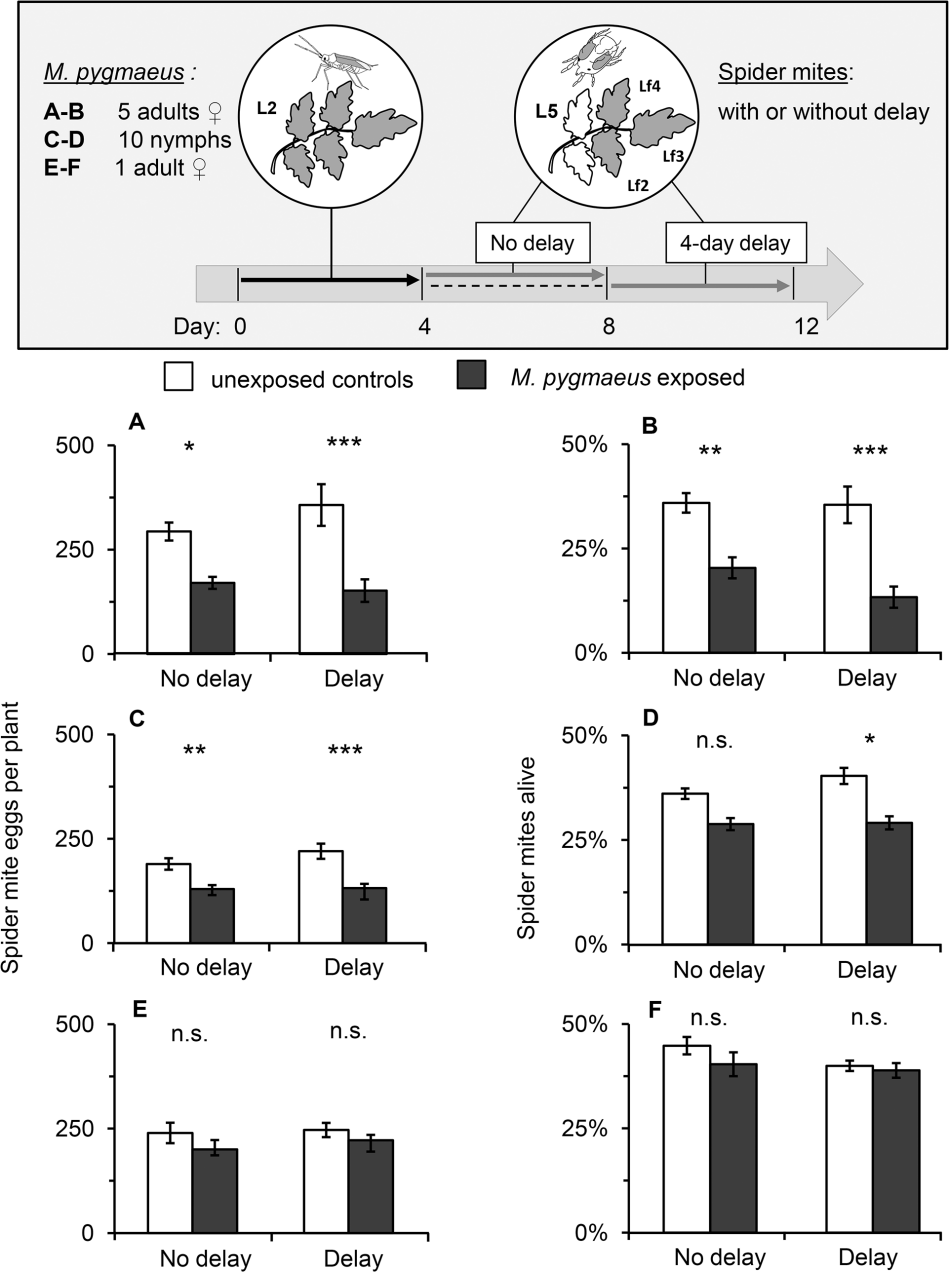
Systemic plant-mediated effects of *Macrolophus pygmaeus* on *Tetranychus urticae*. One or 5 young females or 10 nymphs of *M*. *pygmaeus* were introduced in cages on the second oldest leaf (L2) for 4 days. On younger leaves (L5), 10 young *T*. *urticae* females were kept on each of 3 apical leaflets using lanolin barriers either immediately after exposure (No delay) or with a delay of 4 days (Delay; setup is depicted in inset). (A, C, E) Number of eggs per plant, and (B, D, F) survival of *T*. *urticae* females after 4 days on tomato plants (mean +/- SE, N = 9–11) that were previously exposed to different numbers and stages of *M*. *pygmaeus* (dark bars) or were unexposed (white bars). Significant differences between exposed plants and control plants are indicated by asterisks by Tukey contrasts following a GLMM: n.s.: not significant, *P* < 0.05 (*), *P* < 0.01 (**), *P* < 0.001 (***).

Exposure of plants to *M*. *pygmaeus* also affected spider mite performance on non-exposed leaves of exposed plants (Fig 3). This effect was significant for the number of eggs and live spider mites on leaves three positions higher than the leaf exposed to either 5 *M*. *pygmaeus* females (Fig 3A; GLMM; eggs/plant: χ^2^ = 21.08, df = 3, *P* < 0.001 and Fig 3B; GLMM; mite survival: χ^2^ = 30.48, df = 3, *P* < 0.001) or 10 nymphs (Fig 3C; GLMM; eggs/plant: χ^2^ = 28.29, df = 3, *P* < 0.001 and Fig 3D; mite survival: χ^2^ = 8.96, df = 3, *P* = 0.029), but not when leaves were exposed to 1 *M*. *pygmaeus* female (Fig 3E; GLMM; eggs/plant: χ^2^ = 4.60, df = 3, *P* = 0.203 and Fig 3F; GLMM; mite survival: χ^2^ = 2.27, df = 3, *P* = 0.519). The number of spider mite eggs per plant after exposing lower leaves to 5 *M*. *pygmaeus* females or 10 nymphs was significantly lower than on control plants and this systemic effect was found both directly after exposure of plants to *M*. *pygmaeus*, and with a 4-day delay (Fig 3A and 3C). Furthermore, significantly fewer spider mites were found alive on unexposed leaves of tomato plants previously exposed to 5 females (Fig 3B) or 10 nymphs of *M*. *pygmaeus* (Fig 3D) than on unexposed control plants.

**Fig 4:**
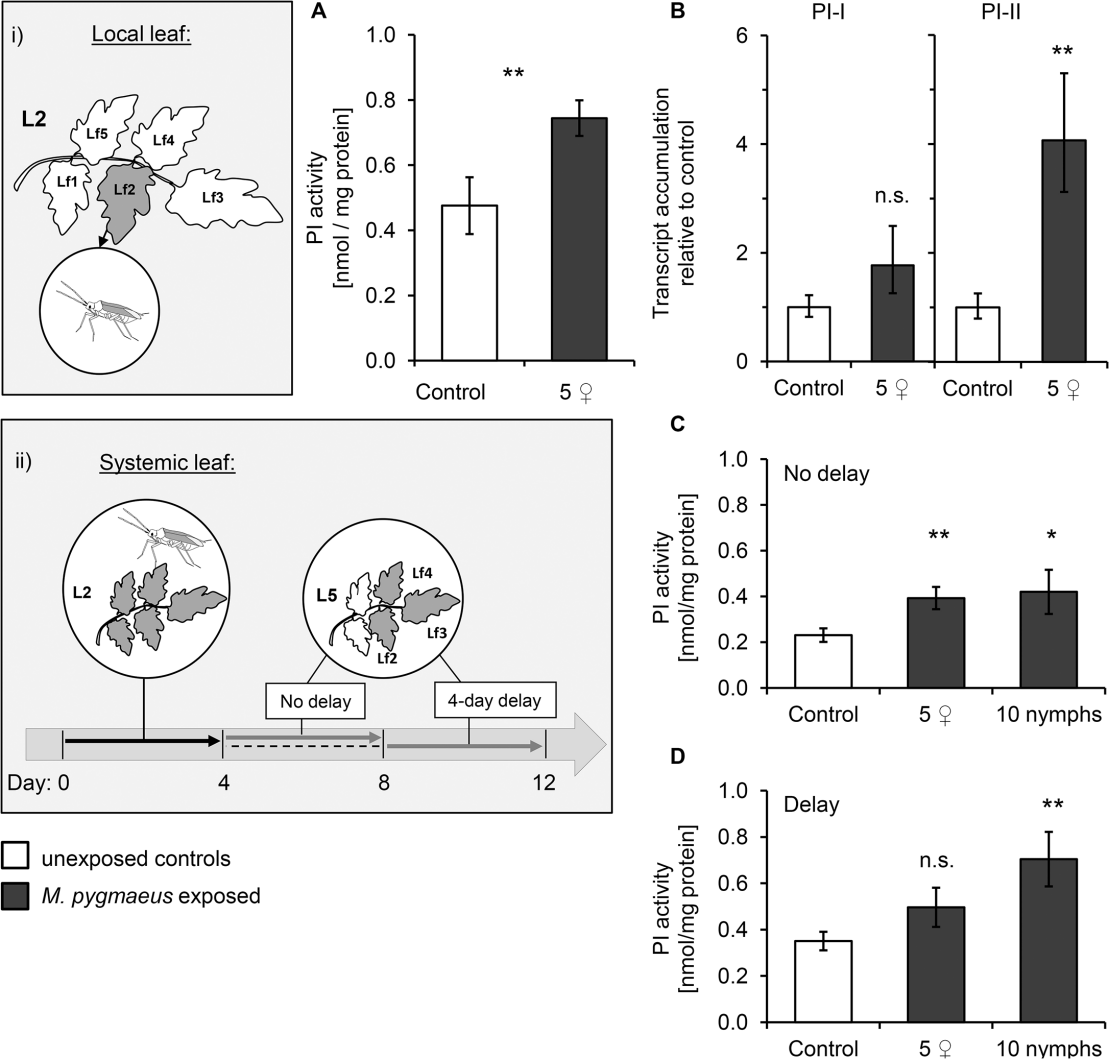
Local and systemic effects of plant exposure to *Macrolophus pygmaeus* on tomato proteinase inhibitor levels. Mean (+/- SE) of (A) proteinase inhibitor (PI) activity relative to protein content (N = 18/19 for control/exposed plants) and (B) transcript accumulation of *PI-I* and *PI-II* genes (N = 5/6 for control/exposed plants, each replicate representing 3 plants) in the local leaflet (Lf) exposed to 5 young *M*. *pygmaeus* females (dark bars) in clip cages for 4 days (depicted in inset i) and in unexposed control plants (white bars). Transcript accumulation expressed relative to control plants was assessed by real time PCR in relation to ubiquitin as reference gene. (C, D) PI activity relative to protein content (mean +/- SE, N = 10/9 for control/exposed plants) of a systemic leaf (L5, pooled Lf 2–4) either directly after (C: No delay) or 4 additional days after (D: Delay) a 4-day exposure of an older leaf (L2), to either 5 young adult females or 10 nymphs of *M*. *pygmaeus* (depicted in inset ii). Significant differences between exposed and control plants are indicated by asterisks following a GLM: n.s.: not significant, *P* < 0.05 (*), *P* < 0.01 (**).

Compared to untreated control plants, PI activity increased by almost 60% in the leaflets exposed to 5 females for 4 days (Fig 4A; GLM; F_1,35_ = 12.54, *P =* 0.001), which matched the accumulation of transcripts of the tomato *PI* genes (Fig 4B; GLM; *PI-I*: F_1,9_ = 1.85, *P =* 0.207; *PI-II*: F_1,9_ = 15.33, *P =* 0.004). Proteinase inhibitor activity also increased in unexposed leaves of plants of which a lower leaf was exposed to *M*. *pygmaeus* (Fig 4C; GLM; 5 females: F_1,17_ = 9.97, *P =* 0.005; 10 nymphs: F_1,17_ = 5.58, *P* = 0.030). Moreover, PI activity was significantly affected even after a 4-day delay subsequent to the exposure to *M*. *pygmaeus* in plants exposed to 10 nymphs compared to unexposed plants (Fig 4D; GLM; 5 females: F_1,18_: 2.14; *P* = 0.160; nymphs: F _1,18_: 8.57; *P* = 0.009). Transcript accumulation of *PI-I* and *PI-II* genes in unexposed leaves harvested without delay was significantly affected by the previous exposure of lower leaves to *M*. *pygmaeus* nymphs (S1 Table; GLM; *PI-I*: F_1,8_ = 5.62, *P =* 0.045, *PI-II*: F_1,8_ = 11.72, *P =* 0.009). However, no significant effects on the accumulation of transcripts of *PI* genes were detected in leaves harvested after a 4-day delay following *M*. *pygmaeus* exposure (S1 Table).

Altogether, these results denote that the plant-mediated effects of the omnivore on spider mites depend on the abundance of predators, and that these effects are not only expressed locally, but also systemically. Furthermore, the decreased spider mite performance largely matched the increased leaf PI activity.

### Persistence of plant-mediated effects

**Fig 5:**
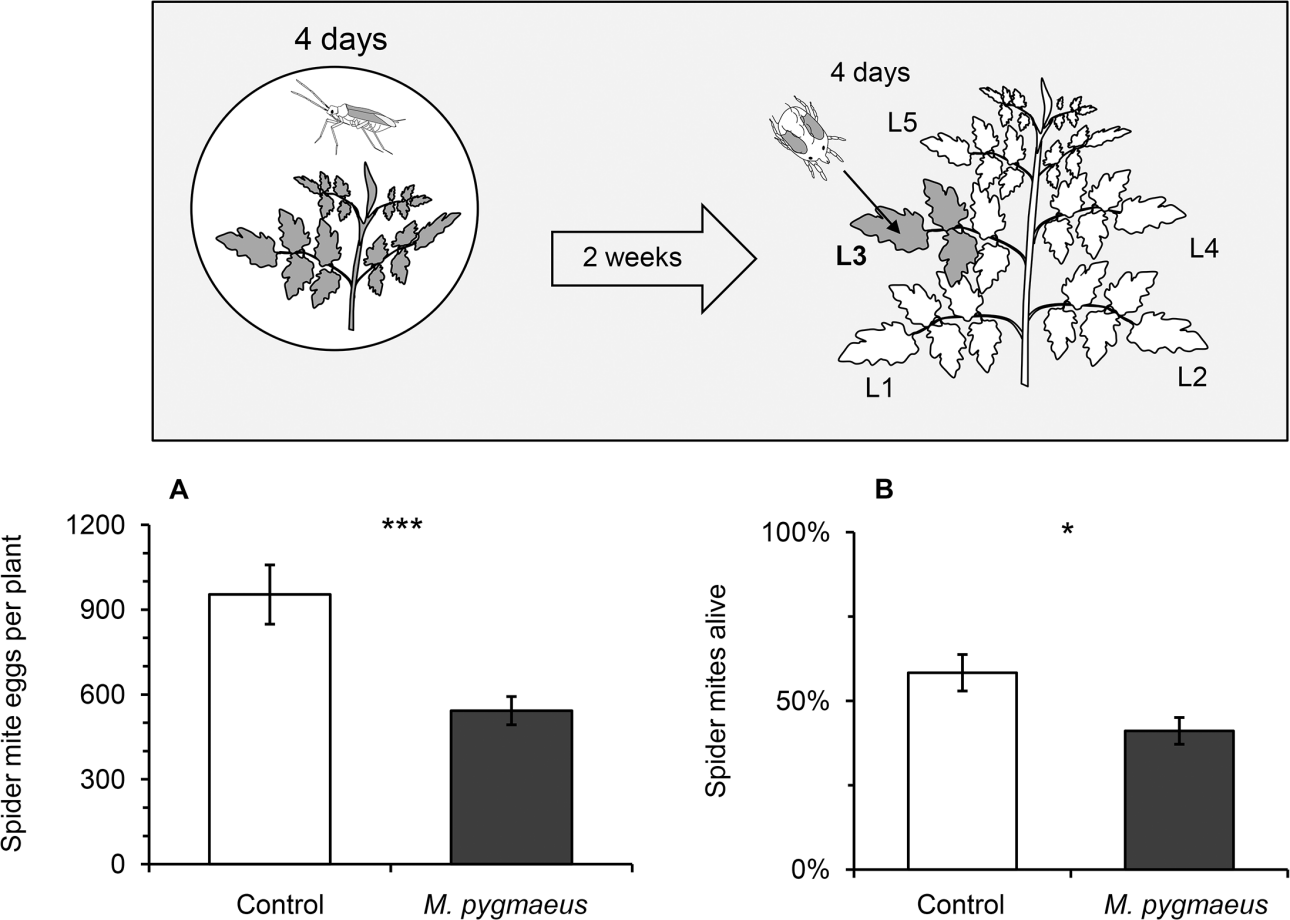
Persistence of plant-mediated effects of *Macrolophus pygmaeus* on the performance of *Tetranychus urticae*. (A) Number of eggs per plant and (B) survival of females of *T*. *urticae* (mean +/- SE of 8 plants) 2 weeks after exposure of young plants to 2 *M*. *pygmaeus* females for 4 days (dark bars, setup is depicted in inset) or the corresponding control plants (white bars). Significant differences between exposed plants and control plants are indicated by asterisks following a GLMM: *P* < 0.05 (*), *P* < 0.001 (***).

Spider mite performance was negatively affected even two weeks after the exposure of young tomato plants to only 2 virgin females of *M*. *pygmaeus* for a period of 4 days (Fig 5). The number of spider mite eggs per plant as well as the number of individuals remaining alive on each plant was significantly reduced on tomato plants exposed to this low density of virgin female predators two weeks earlier (Fig 5A; GLMM; eggs/plant: χ^2^ = 11.57, df = 1, *P* < 0.001 and Fig 5B; GLMM; mite survival: χ^2^ = 4.01, df = 1, *P* = 0.045).

## Discussion

Plant responses induced by herbivores have been well investigated in numerous plant systems; however, little is known of the response of plants to feeding by omnivorous predators. Our data show that an omnivore displays plant-mediated negative effects on the performance of one of its herbivorous prey. Moreover, we report that plants respond to the phytophagy by omnivorous predators in that they show increased levels of proteins involved in plant defence, suggesting that other tomato pests sensitive to these defences may also be negative affected.

The performance of two-spotted spider mites on tomato plants that had been previously exposed to *M*. *pygmaeus* was not only reduced on the leaf that was exposed to the predator but also on systemic leaves, suggesting that this reduction was not due to a response to kairomones derived from the predator. In a similar study, oviposition by the omnivorous predator *O*. *laevigatus* on tomato, which causes wounds in the plant, was found to induce expression of defence-related genes and to reduce feeding damage by thrips [25]. However, it is unclear whether the reduced feeding was caused by a response of thrips to altered plant quality or to other cues associated with this omnivorous predator. In our study, the exposure of tomato plants to *M*. *pygmaeus* induced a plant response similar to herbivore-associated damage. Also, exposure of plants to young adult females, nymphs and young virgin females of the predators had fairly similar effects on spider mite performance and PI induction. Thus, it is likely that phytophagy by *M*. *pygmaeus* induced plant resistance. Oviposition by herbivores is known to induce plant defence responses [4], therefore an additional effect of oviposition by mirid females cannot be excluded although care was taken to use young adults during their pre-oviposition period. However, plant-mediated effects shown in our study did not require oviposition by *M*. *pygmaeus* as they were also observed after plant exposure to juveniles which is also in line with an increased transcript accumulation of the *PI-II* gene in response to plant feeding by the zoophytophagous predator *N*. *tenuis* on tomato [26].

The specific way in which *M*. *pygmaeus* feeds on plants has not yet been investigated, but other zoophytophagous hemipterans, such as *Orius insidiosus* and pentatomids feed on plants mainly to acquire water from the xylem and potentially also nutrients from the mesophyll, thereby probably causing some minor cell wounding [32–34]. In contrast to these predators, mirids, such as *M*. *pygmaeus*, are assumed to be able to dilacerate plant tissue and also produce saliva with pectinases that are essential enzymes for phytophagy [27, 35]. Therefore, *M*. *pygmaeus* probably induces wound responses in plants, as do other cell-content feeders. In tomato, this wound response was first discovered as induced manifestation of PI activity upon wounding and herbivory [1], which in turn is due to transcriptional up-regulation of two serine PI genes mediated by the JA signalling pathway [36]. In line with elicitation of the tomato wound response upon phytophagy, we observed induced PI activity and transcriptional up-regulation of the same *PI* genes as mentioned above, although up-regulation was only significant for the *PI-II* gene. In agreement with earlier results on the tomato wound response [37], we also found a local and systemic induction of PI activity. However, it remains unclear how specific the response of tomato plants is to phytophagy by *M*. *pygmaeus*. Wound-induced responses of plants are often modified by perception of herbivore-specific elicitors (e.g. [2]), which may also be the case for zoophytophagous omnivores, especially within the Miridae, as they produce many different salivary enzymes [27, 35].

The decreased performance of *T*. *urticae* largely matched with the induction of PI activity, suggesting that plant defence traits induced through the tomato wound response may have caused this reduction. Earlier work also suggests that *T*. *urticae* can be directly affected by the wound response induced by spider mite feeding on tomato plants because it performs better on *def-1* mutants that are deficient for JA biosynthesis than on wild type plants [38, 39]. However, it remains unclear which induced direct defence traits are active against spider mites and to which extent the induced PI activity contributes to the negative effect on spider-mite performance. Although there is solid evidence for the defensive function of solanaceous serine PIs against generalist and specialist lepidopteran herbivores and sucking phytophagous mirids [40–42], their efficiency against phytophagous mites still needs to be determined and spider mites are known to vary in their susceptibility to JA defences [43, 44]. Since it was technically not possible to daily count the number of live spider mites on intact plants, and therefore calculate daily oviposition per female, we cannot distinguish among antixenosis and/or antibiosis effects against spider mites caused by *M*. *pygmaeus*. Therefore, further research is required to functionally link the JA mirid-induced responses to the decreased performance of spider mites shown in our study and to determine how *T*. *urticae* may affect the plant’s response to feeding by *M*. *pygmaeus*. On the other hand, *M*. *pygmaeus* feeding may have decreased the plant’s nutritional quality, which could explain the decrease in spider mite performance. However in such a case, lower plant quality should have affected both spider mites and whiteflies.

In contrast to its effect on spider mites, plant exposure to *M*. *pygmaeus* did not affect the performance of the whitefly *T*. *vaporariorum* infesting these plants directly after *M*. *pygmaeus* had been removed. Possibly, the defensive capabilities of the JA-mediated plant defences against these two herbivores differ. Whiteflies are phloem-feeders, many of which circumvent wounding of plant tissue [45] by inducing the transcription of SA-related genes while not inducing transcription of JA-related genes in various plant species [16, 46–48]. Moreover, whiteflies may also not be very susceptible to JA-mediated defences [49]. Although studies that directly test the effect of JA-mediated tomato defences on *T*. *vaporariorum* are lacking, *B*. *tabaci* and *T*. *vaporariorum* are known not to activate wound-responses [46, 50]. No effect on *B*. *tabaci* performance in the absence of the endogenous signal (spr2 mutant) was found using jasmonate-deficient *spr2* tomato mutants altered in systemin-signalling, which is involved in JA-signalling and thus also in *PI* gene activation [51]. However, nymphal development of *B*. *tabaci* on tomato plants constitutively overexpressing pro-systemin was diminished suggesting that whitefly juvenile development can be negatively affected if JA-mediated defences are massively activated [16, 51]. Thus, it is relevant to investigate whether phytophagy by *M*. *pygmaeus* affects whitefly nymphal development or whether whiteflies are capable of suppressing or withstanding plant defences activated by the predator.

The fact that zoophytophagous predators can induce direct plant defences against herbivory raises many questions on the ecological implications of this phenomenon. An important first question is, whether the plant evolved to express a specific response to the phytophagy of *M*. *pygmaeus* or whether it is a response that evolved in the plant’s interaction with herbivores, such as phytophagous mirids. Phytophagy of predators should be tolerated by the plant because it benefits from the presence of the predators as a defence component against herbivore invasions. However, when herbivores are permanently absent, the benefits of maintaining predatory insects on plants are abolished and the costs of their plant feeding may even increase. Therefore, herbivore-free plants may benefit from defending against the feeding damage by zoophytophagous insects, though it remains to be determined whether the plant responses induced by *M*. *pygmaeus* are capable of reducing its phytophagy or if *M*. *pygmaeus* has evolved specific mechanisms to overcome or avoid plant defences [52, 53]. The plant’s response to zoophytophagous predators may even depend on the concomitant presence and/or abundance of herbivores. Thus, it would be interesting to unravel, whether and how the plant responses to zoophytophagous predators and to its herbivorous prey are affected by the concomitant or sequential occurrence of the other.

We conclude that zoophytophagous predators can affect herbivorous prey directly through predation, but also indirectly via plant-mediated effects. Our data support the hypothesis that these plant-mediated effects involve the induction of plant defences in response to the phytophagy by omnivorous insects. At first sight, phytophagy by predators may be detrimental to plants, but provided it results to lower prey populations and respective damage—as our study shows—it may also benefit plants when threatened by herbivory because it can activate direct plant defences in addition to promoting the persistence of zoophytophagous predators on the plant. If so, mild phytophagy by predators may also ‘vaccinate’ plants against herbivore attack later in the season (as shown for genuine herbivores (e.g. by [5, 12, 54]). In this respect it is interesting to recall that we observed a relatively long-lasting and systemic resistance of tomato plants against spider mites after exposure to a zoophytophagous predator. Pre-plant application of zoophytophagous predators, a method suggested for zoophytophagous mirids to faster establish in the crop (e.g. [55]), may thereby increase the direct defence of tomato against herbivory. This creates a new perspective for the use of the omnivorous predator *M*. *pygmaeus* as both a generalist biological control agent already widely used for directly suppressing populations of plant pests such as spider mites, aphids and whiteflies [27], and as an agent affecting plant resistance to forthcoming pests. Our study highlights a neglected feature of omnivory that should be considered in biological pest control.

## Supporting Information

S1 Table Systemic effects of plant exposure to *Macrolophus pygmaeus* on tomato transcript accumulation of *PI-I* and *PI-II* genes. (DOCX)
