# Supplementary material for: Beyond Predation: The Zoophytophagous Predator Macrolophus pygmaeus Induces Tomato Resistance against Spider Mites
Source: PLoS One. 2015 May 14;10(5):e0127251. doi: 10.1371/journal.pone.0127251 (PMC4431799; doi:10.1371/journal.pone.0127251)
Supplement: S1 Table — (DOCX) [file pone.0127251.s001.docx]

**Supporting Information**

**Supporting Information Table S1. Systemic effects of plant exposure to *Macrolophus pygmaeus* on tomato transcript accumulation of *PI-I* and *PI-II* genes.**

| **Systemic leaves after exposure to *M. pygmaeus*** | | | | | | | | | | | | |
| --- | --- | --- | --- | --- | --- | --- | --- | --- | --- | --- | --- | --- |
|  | **none (Control)** | | |  |  | **5 adults** | | |  | **10 nymphs** | | |
| **A** |  | | |  |  |  | | |  |  | | |
| **PI-I** |  |  |  |  |  |  |  |  |  |  |  |  |
| Expression | 1.00 | +/- | 0.35 |  |  | 0.94 | +/- | 0.16 |  | 1.93 | +/- | 0.51 |
| *P*-value |  | | |  |  | *P* = 0.553 | | |  | ***P* = 0.045*** | | |
| **PI-II** |  |  |  |  |  |  |  |  |  |  |  |  |
| Expression | 1.00 | +/- | 0.40 |  |  | 0.99 | +/- | 0.26 |  | 3.26 | +/- | 0.51 |
| *P*-value |  | | |  |  | *P* = 0.504 | | |  | ***P* = 0.009**** | | |
| **Systemic leaves after a 4-day delay after exposure to *M. pygmaeus*** | | | | | | | | | | | | |
|  | **none (Control)** | | |  |  | **5 adults** | | |  | **10 nymphs** | | |
| **B**  **PI-I** |  |  |  |  |  |  |  |  |  |  |  |  |
| Expression | 1.00 | +/- | 0.39 |  |  | 1.20 | +/- | 0.20 |  | 1.11 | +/- | 0.25 |
| *P*-value |  | | |  |  | *P* = 0.311 | | |  | *P* = 0.460 | | |
| **PI-II** |  |  |  |  |  |  |  |  |  |  |  |  |
| Expression | 1.00 | +/- | 0.26 |  |  | 2.69 | +/- | 0.87 |  | 1.44 | +/- | 0.51 |
| *P*-value |  | | |  |  | *P* = 0.077 | | |  | *P* = 0.721 | | |

Transcript accumulation of *PI-I* and *PI-II* genes (mean +/- SE, N = 5, each replicate represents a pooled sample of 2 plants) in leaves L5 (leaf positions are depicted in inset ii of Fig.4) of tomato plants that were either exposed to 5 adults, or 10 nymphs of *M. pygmaeus* on a lower position (L2), or were left unexposed (control). Transcript accumulation was measured by real-time PCR analysis relative to ubiquitin as reference gene and is displayed relative to control plants. Plants were sampled directly either after being exposed to *M. pygmaeus* for 4 days (A) or after a delay of 4 additional days (B) without the omnivore, *P* < 0.05 (*), *P* < 0.01 (**).
